# Fe(II)-phthalocyanine supported on chitosan aerogel as a catalyst for oxidation of alcohols and alkyl arenes

**DOI:** 10.1038/s41598-021-03226-7

**Published:** 2021-12-09

**Authors:** Fatemeh Azimi, Ahmad Poursattar Marjani, Sajjad Keshipour

**Affiliations:** 1 Department of Organic Chemsitry, Faculty of Chemistry, Urmia University, Urmia, Iran; 2 Department of Nanotechnology, Faculty of Scince, Urmia University, Urmia, Iran

**Keywords:** Catalysis, Green chemistry

## Abstract

Manipulation of materials is considered as one of the eminent strategies to create desirable catalysts. In this regard, increasing surface area and decreasing dimensions of catalysts have been widely employed on account of effectiveness of these methods. Herein, aerogel form of chitosan as a sustainable, and high aspect ratio compound is employed as a green support for the catalytic purposes. Chitosan aerogel was modified with Fe(II)-phthalocyanine to produce a heterogeneous catalyst for oxidation reactions. The synthesized catalyst promoted the oxidation reactions of alcohols and alkyl arenes to the corresponding aldehydes and ketones using H_2_O_2_ as an oxidant in 24 h. The reactions for aliphatic and aromatic alcohols gave turnover numbers of 106–109 at 80 °C and 106–117 at room temperature, respectively. The oxidations of alkyl arenes were carried out with turnover numbers laying in the range of 106–117 at 100 °C. The low toxicity, inexpensive nature, and recycling possibility of the catalyst accompanied by the reaction mild conditions, clean oxidant, and excellent yields offer chitosan aerogel modified with Fe(II)-phthalocyanine as a promising catalyst for oxidation reactions.

## Introduction

Phthalocyanines (Pc) are aromatic macrocyclic compounds with the broad applications such as the colorants due to their intense colors. Nowadays, these materials and their metalated complexes (MPc) are the center of attention for the catalyst researchers in position of directing a wide variety of aerobic transformations, reductions, and decomposition of peroxides^1^. In the meantime, MPcs are of importance possessing some unique characteristic, including special optical, and electronic properties^2,3^. The application of this category of multi-cyclic compounds are extending due to their easy preparation approach, significant activity, and high economic efficiency^1^. As a result, recent decade witnesses a great number of reports about MPc especially their applications as catalysts. Fe(II)-Pc is an active MPc that was employed in the oxidation of wide variety of organic substrates including *β*-isophorone^4^, catechol^5^, starch^6^, aromatic pollutant^7^, cyclohexene^8^, cyclohexane^9^, alcohols^10^, and methane^11^. The high activity of this catalyst is attributed to the large number of π electrons which are easily transported between the catalyst and substrate, similar to its analogue, Fe(II)-porphyrin^12^. The significant catalytic activity of MPcs encourage researchers to deposit them on various supports to achieve a high-performance heterogeneous catalyst with some advantages such as conducting the reaction by the low catalyst amounts, and the easy catalyst recycling^13–15^.

Utilization of heterogeneous catalysts are surprisingly increasing since they could tackle the environmental concerns created by catalytic procedures^16^. The kind of support for these catalysts received great attention and among them, biocompatible ones obtained priority as they make lower pollutions^17–19^. Furthermore, the support can afford more benefits to the catalysts such as a high surface area to boast its activity^20^. Therefore, natural-based materials in their porous forms such as chitosan aerogel (ChA) are the best supports. Besides, chitosan having numerous amines is susceptible to be modified by electrophilic groups containing materials^21^. However, there are a few numbers of catalysts supported on ChA such as Dimaval-Ru(III)^21^, Pd(II)-Pc^22^, and dimercaprol-Au (III)^23^.

Superb activity and broad range of the applications of Fe(II)-Pc especially in the oxidation reactions directed us to deposit this active catalyst on a sustainable support. We believe that ChA was a promising support for our agenda due to the nontoxicity, biocompatibility, availability, low cost, and high surface area^21,23^. Therefore, we modified ChA with Fe(II)-Pc (FePc@ChA) to obtain active and biocompatible heterogenous catalyst. The prepared catalyst was employed for the oxidations of aliphatic and aromatic alcohols, and alkyl arenes.

## Experimental

### General methods

Chemicals were acquired from Merck and Aldrich and used with no subsequent purification. Fe(II)-phthalocyanine-4,4′,4″,4‴-tetrasulfonic acid was provided from Sigma-Aldrich. Scanning Electron Microscopy (SEM), Energy Dispersive X-ray Spectroscopy (EDX), and elemental mapping were performed by Scanning Electron Microscope of TSCAN Company. Characterization of the functional groups was carried out by Fourier transform infrared spectroscopy (FT-IR) using an AVATAR FTIR instrument of Thermo company in the range of 400–4000 cm^-1^. Elemental detections were accomplished using a Flame Atomic Absorption Spectroscopy (FAAS) (Shimadzu AA-680). Brunauer–Emmett–Teller analysis was done by Belsorp mini II instrument of Microtrac Bel Corp at 77 K after drying the sample under reduced pressure for 24 h. Thermal gravimetric analysis (TGA) was carried out by a Linseis STA PT1000. Gas chromatography (GC) was performed in Varian 3900 GC with 260 °C of injector and detector temperature, 100 and 280 °C for initial and final temperatures respectively, and the temperature ramp was adjusted on 3 °C/min.

### Preparation of FePc@ChA

For the preparation of ChA, a solution of chitosan was prepared by solving 2 g of the polymer in acetic acid solution (50 mL, 1.00 vol%) under sonication. Next, glutaraldehyde (2 mL) was added to the solution under vigorous stirring for 10 min to prepare a gel and the obtained product was kept for 24 h. Then, the gel was immersed in ethanol at room temperature to eliminate the unreacted acetic acid and aldehyde. After that, freeze-drying technique at − 40 °C was used to obtain the desired aerogel after pouring obtained mixture into 200 ml of distilled water^21^.

Fe(II)Pc-4,4′,4′′,4′′′-tetrasulfonic acid (0.20 g) and ChA (2.00 g) was stirred in H_2_O (10 ml) at 70 °C for 24 h. Then, the mixture was filtered off, the residue was added to HCl (0.1 N, 20 ml), and the obtained mixture again filtered off after 0.5 h stirring to obtain FePc@ChA. The FePc@ChA was again purified by the mentioned protocol and then, the product was dried in an oven at 80 °C.

### Fe determination on FePc@ChA with AAS analysis

HCl:HNO_3_ mixture (3:1) was prepared and FePc@ChA (0.1 g) was added to the 10 ml of the prepared mixture under sonication for 3 h. Next, the residue was filtered off, and the remained solution was treated by distilled water to increase the volume up to 20 ml. Finally, the solution was analyzed by AAS to determine Fe concentration using calibration curves prepared by standard solutions of the metal cation.

### Typical procedure for the oxidation of benzyl alcohol with FePc@ChA

To a round-bottomed flask containing benzyl alcohol (10 mmol) and FePc@ChA (0.2 g), 11 mmol of H_2_O_2_ was added dropwise under mechanically stirring at room temperature. Thin-layered chromatography determined the reaction end. After 24 h, FePc@ChA was filtered out and the solution was analyzed by GC. Turnover numbers were determined by the following equation:

$${\text{TON}} = {\text{mmol of the desired product}}/{\text{mmol of FePc}}$$

## Results and discussion

**Scheme 1:**
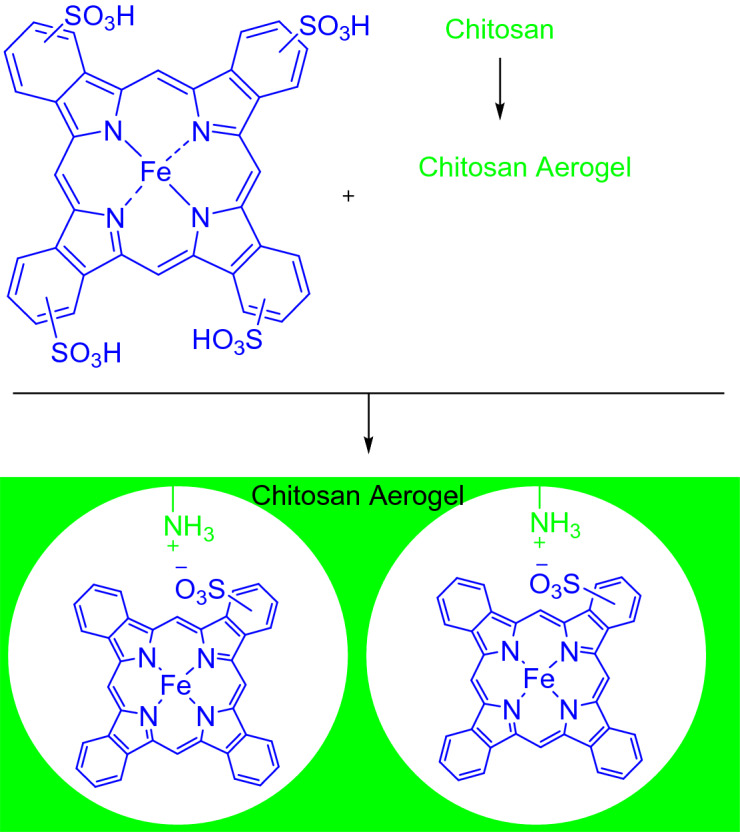
Synthesis of FePc@ChA.

As a matter of fact, ChA provides high surface area for the progress of the catalytic reactions, leading to the better catalyst performance. In addition, abundant amine groups in the ChA structure makes it susceptible for emendation with a wide variety of functionalities. The simplest conceivable reaction for the amines of ChA could be the acid–base reaction with an acid like FePc-4,4′,4″,4‴-tetrasulfonic acid. The reaction was progressed by a proton transfer from the sulfonic functionality to amine, leading to the formation of ammonium on the ChA and sulfonate on the FePc. The opposite formed charges created strength ionic interactions between ChA and FePc to obtain FePc@ChA as a heterogeneous catalyst (Scheme 1).

**Figure 1:**
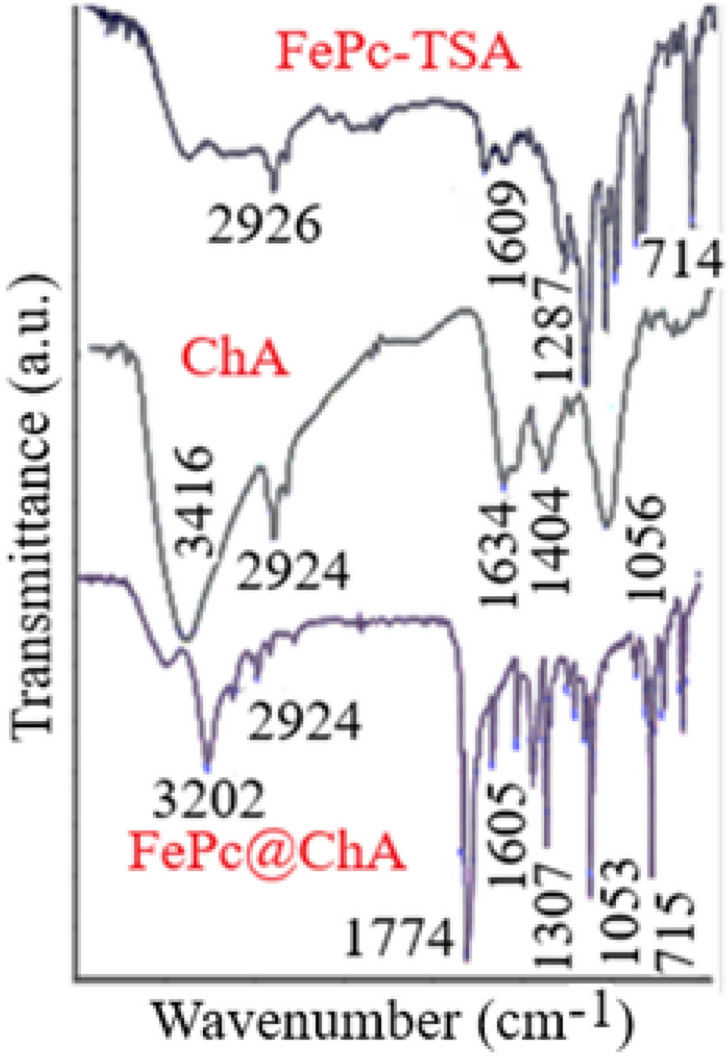
FT-IR spectra for FePc, ChA, and FePc@ChA.

The successful deposition of FePc on ChA was confirmed by FT-IR spectra. FePc@ChA showed peaks attributed to Pc and ChA (Fig. 1). Bands at 3202, 2924, and 1053 cm^-1^ in the FePc@ChA spectrum are ascribed to the stretching vibrations of OH, CH, and C–O groups of ChA, respectively. The peak at 1609 cm^-1^ was attributed to the stretching vibration of C = N groups of Pc. The sulfonic groups showed a peak at 1307 cm^-1^ for the assymetric stretching vibration of S = O, and another peak at 715 cm^-1^ for the stratching vibration of S-C groups.

**Figure 2:**
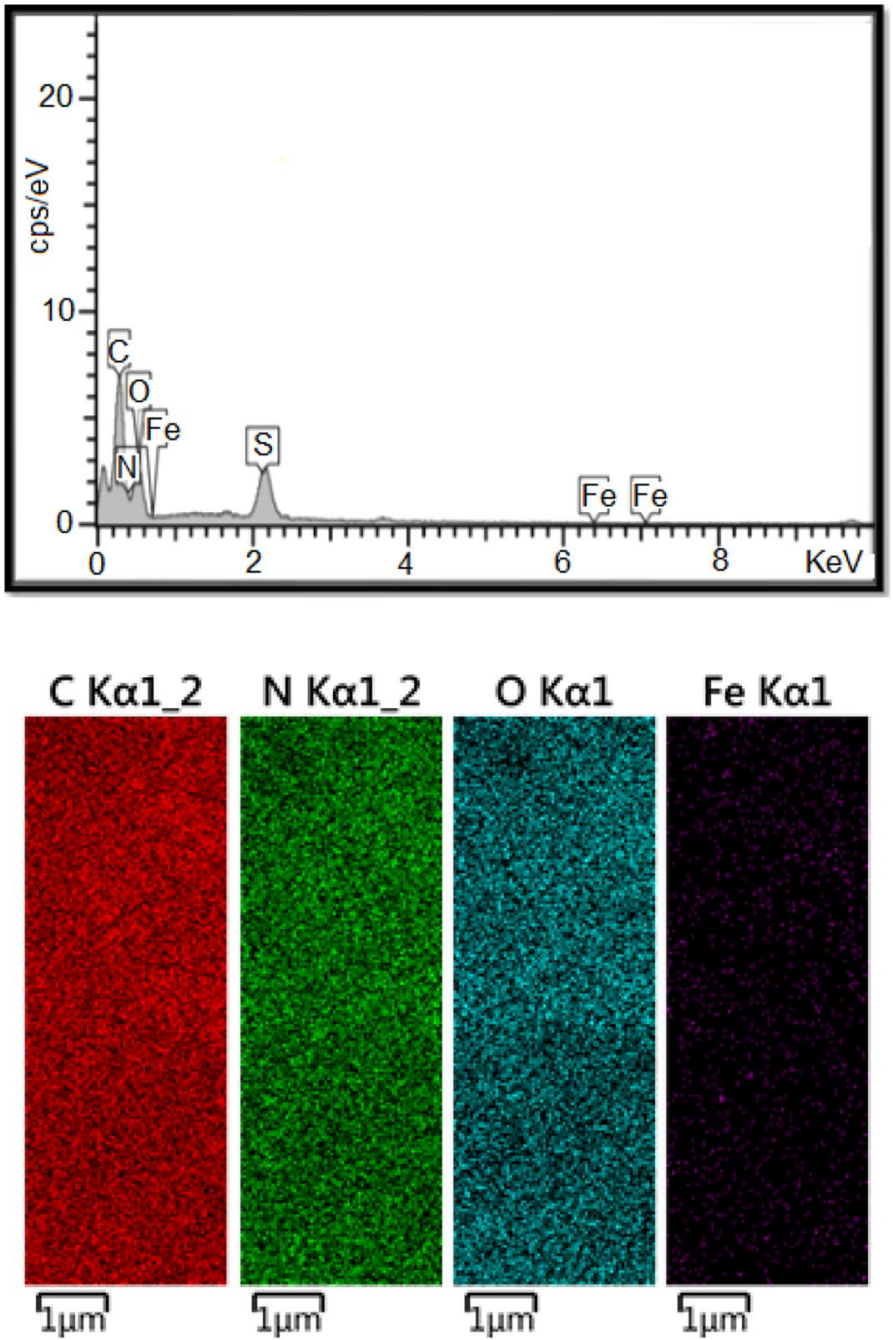
EDX microanalysis and elemental mapping on FePc@ChA.

The catalyst surface was investigated with EDX. The analysis of FePc@ChA confirmed incorporation of Fe, N, and S atoms in the catalyst structure, approving triumph loading of FePc on ChA. The Fe concentration was 0.036 mmol/g of FePc@ChA measured by FAAS. Moreover, elemental mapping was carried out on FePc@ChA to determine FePc distribution on ChA since uniform loading of a catalyst on the support improves its catalytic activity. The Fe atoms distribution on the composite demonstrated homogeneous perching of FePc on the support (Fig. 2).

**Figure 3:**
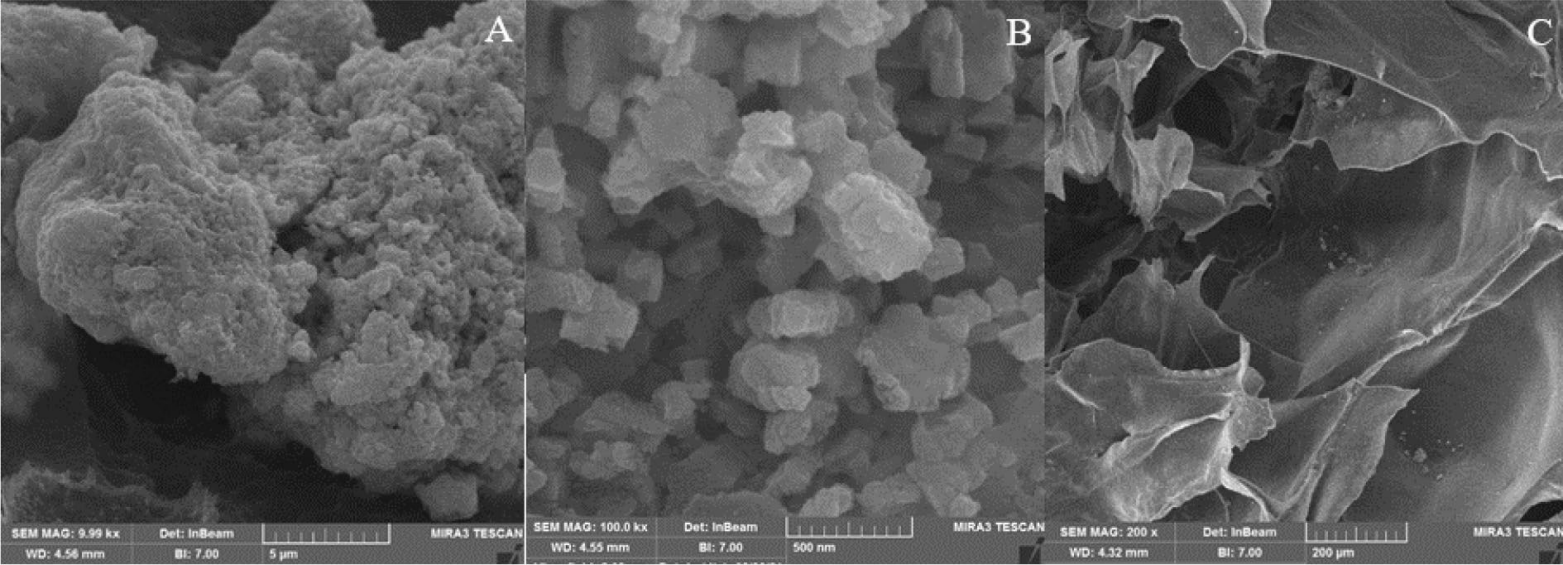
SEM images of ChA (**A** and **B**) and FePc@ChA (**C**).

Microscopic studies on ChA and FePc@ChA was carried out to observe the porous structures and the changes during the modification. The SEM micrograph for ChA showed a big porous particles (Fig. 3A), in which focusing on the observed pieces revealed a fine particles aggregated in a porous structure (Fig. 3B). The SEM micrographs also showed crystalline structure of FePc@ChA, indicating a porous polymer in whole the sample (Fig. 3C)^23^. These results confirmed that the porosity of the ChA as a significant factor for a support was retained during the chemical modification.

**Figure 4:**
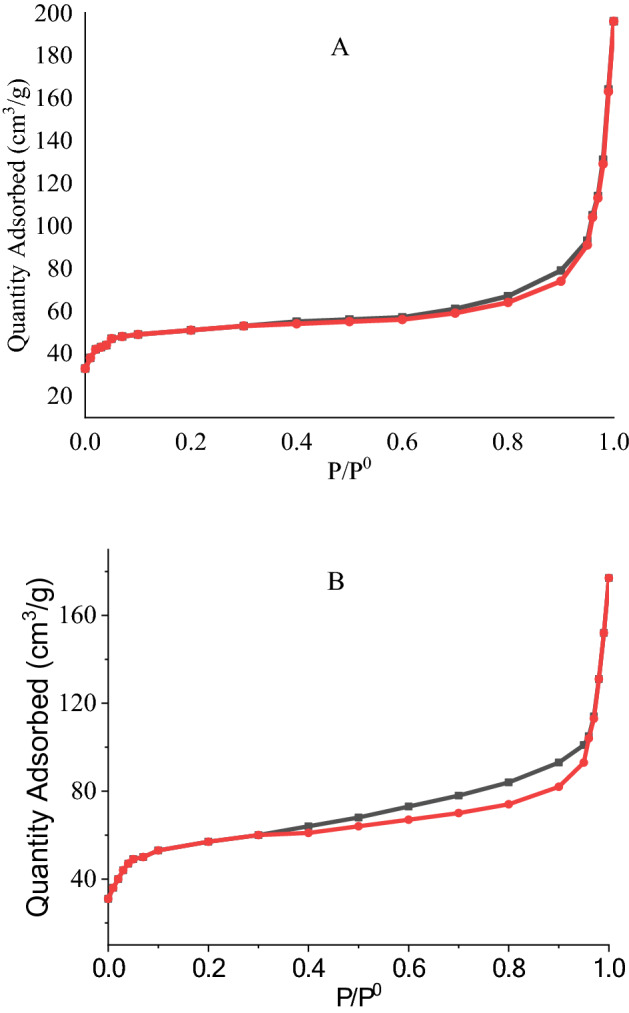
Adsorption–desorption isotherms of ChA (**A**) and FePc@ChA (**B**).

Isotherms of adsorption–desorption for ChA and FePc@ChA were prepared at 77 K, after drying the samples under vacuum at 100 °C for 24 h (Fig. 4). Isotherms for both samples demonstrated macroporous structures with the diagram of type II, in which the surface areas were calculated to be 156 and 148 m^2^.g^-1^ for ChA and FePc@ChA, respectively. These results indicated that the modification reaction slightly decreased the surface area, but this value is not concerning. Furthermore, the isotherms of adsorption and desorption for FePc@ChA indicated some differences related to closing some of the pores in the ChA structure.

**Figure 5:**
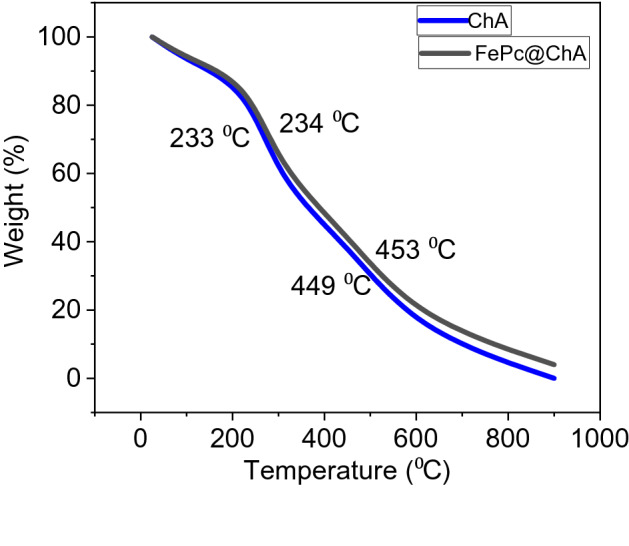
TGA curves of ChA and FePc@ChA.

Thermal Gravimetric Analysis on FePc@ChA was employed to study its thermal stability in the air and evaluate the modifications impressions on thermal behavior of ChA (Fig. 5). The curve of FePc@ChA evinced a main weight loss at 234 °C attributed to the polymeric structure breaking into small units^23^. A similar weight-loss was observed for ChA, in which about 40% of the weight was loosen. Another weight-loss was occurred at about 450 °C for both ChA and FePc@ChA, related to complete decomposition of the ChA structure and releasing gasses^23^. After this deconstruction, the remained FePc of FePc@ChA was decomposed at about 615 °C^24^.

**Figure 6:**
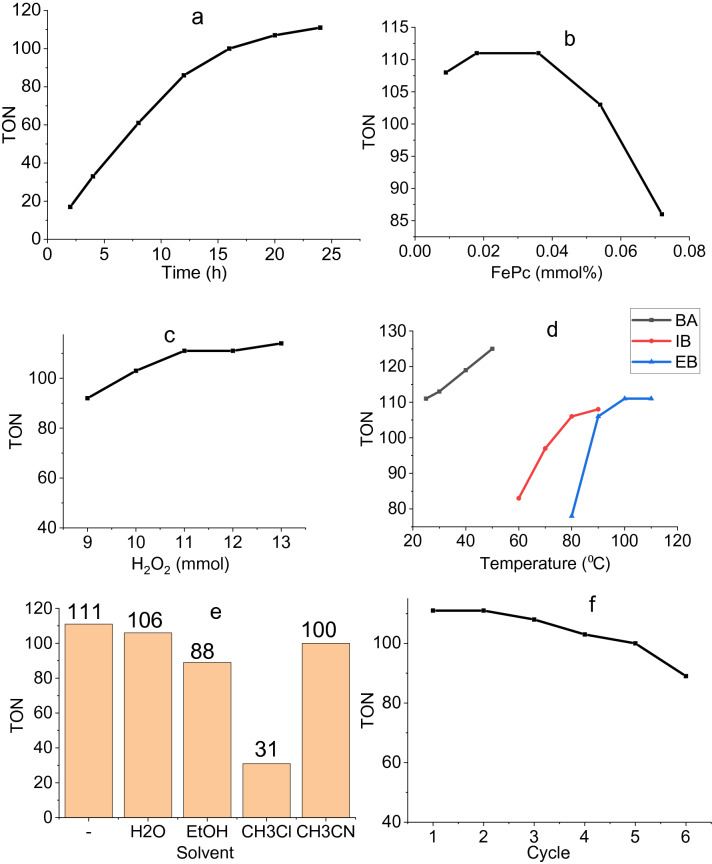
(**a**) TON vs time for oxidation of BA, (**b**) effect of FePc amounts on the oxidation of BA, (**c**) effect of H_2_O_2_ amounts on the oxidation of BA, (**d**) temperature effect on oxidation reaction of BA, IB, and EB; (**e**) solvent effect on oxidation of BA; (**f**) recyclability of catalyst in the oxidation of BA.

FePc@ChA was employed as a heterogeneous catalyst in the oxidation reactions of alcohols, and alkyl arenes using H_2_O_2_ as an oxidant. The catalyst was tailored for use in the oxidation of benzyl alcohol (BA) by H_2_O_2_ at room temperature for 24 h. The reaction afforded 40% conversion of benzaldehyde using 0.036 mmol of FePc, in which Turnover Number (TON) was calculated to be 111. Screening TON during the time revealed high conversions at the beginning and decreasing the rate especially after 12 h (Fig. 6a). FePc loading impressed the reaction yield as the best TON was obtained by 0.018–0.036 mmol of the catalyst (Fig. 6b). Additional increasing in the catalyst amounts decreased the TON since high numbers of the catalysts interfering together. Oxidant amounts affected the reaction yields, in which 11 mmol of H_2_O_2_ was found as the best amount (Fig. 6c). To assay the possibility of using aerobic conditions, the reaction was evaluated by an oxygen atmosphere, in which the yield did not satisfy us with 1.8% conversion. As a rule, increasing the temperature could elevate the reaction yield and its positive effect was observed in the oxidation of BA (Fig. 6d). The temperature influence was also studied on the oxidation of isobutyl alcohol (IB) and ethylbenzene (EB) at 80 and 100 °C as the optimized ones, respectively. Conducting on the oxidation of BA in various solvents shed light on the fact that solvent-free conditions provide highest TON (Fig. 6e). To clarify ChA and FePc effects on the reaction, the BA oxidation reaction was also examined by ChA and FePc which gave 0.8% and 31% conversion, respectively. Therefore, ChA could not successively promote the reaction in the absence of FePc. These tests also confirmed the catalytic activity of FePc and the role of ChA as a support in the boosting of the FePc activity.

The oxidation reaction was extended using some alcohols, and alkyl arenes (Table 1). Totally, the reaction was easier proceeded for alcohols compared to alkyl arenes. For instance, alcohols oxidations were carried out at lower temperatures than alkyl arenes. Furthermore, among used alcohols, aromatic ones have been oxidized at lower temperatures with higher conversions.

**Table 1:** Oxidation of alcohols and alkyarenes by FePc@ChA^*a*^.

Entry	Substrate	Product	Temperature (°C)	Conversion (%)	TON
1			25	40	111
2			25	38	106
3			25	42	117
4			80	38	106
5			80	37	109
6			80	37	109
7			100	40	111
8			100	38	106
9			100	42	117

^a^Reaction conditions: Substrate (10 mmol), FePc@ChA (0.036 mmol) and H_2_O_2_ (11 mmol), 24 h.

FePc release from FePc@ChA to the reaction media of BA was investigated through a leaching study. For this purpose, after removing the catalyst by filtration, the filtrate was analyzed by FAAS to detect Fe. The analysis did not indicate any Fe since FePc is insoluble and does not leach from the catalyst to any solvent and it was also bonded by strong ionic interactions to the support. In addition, a hot filtration test was conducted to determine the reaction phase. The test was carried out by separation of the catalyst from the reaction mixture after 1 h and the reaction progress was screened, in which the reaction was quenched. This examination demonstrates heterogeneous progressing of the reaction.

A recycling test was done on FePc@ChA to determine its stability in the BA oxidation media (Fig. 6f). In this regard, FePc@ChA was filtered off at the end of the reaction, washed with acetone, and reused. The procedure was repeated 6 times, approving the catalyst stability. As a result, the possibility of recycling FePc@ChA for 6 times permits conducting the reaction by recycled catalyst without significant decreases in the oxidation conversions.

**Scheme 2:**
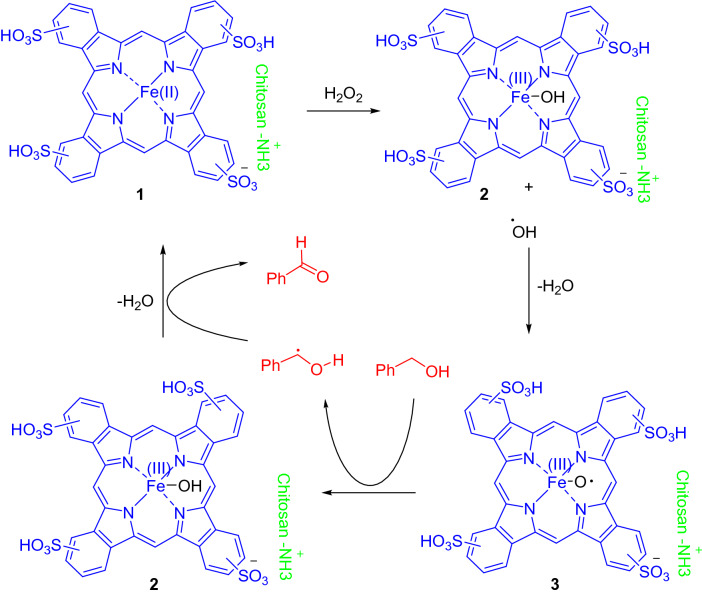
The proposed mechanism for oxidation reaction of BA by FePc@ChA.

It is worthy to note that, Pcs are active compounds in the producing radical species, leading to acceleration of reactions through radical mechanism^25^. Besides, Fe as the core metal increases generation of radicals. Considering this background, we proposed a mechanism for these reactions based on a radicalic approach, which for BA oxidation presented in Scheme 2. The reaction is initiated by the reaction of FePc and H_2_O_2_ to generate Fe(III)Pc-OH (2). Species 2 is activated by the reaction with hydroxyl radical to produce intermediate 3 which absorbs a hydrogen radical from BA to again provide 2. Compound 2, with a hydrogen uptake from radicalic BA furnishes benzaldehyde. This mechanism elucidates the reason for easy oxidation of aromatic alcohols compared to aliphatic ones since the radaicalic intermediates are produced during the reaction are stabilized by the aromatic rings. The stable intermediates offer easier transformations. Furthemore, the alkyl arenes need to be transformed to alcohols and then to the aldehydes or ketones, in which need to the higher temperatures shows that the first step goes hard.

The accomplished oxidation of BA with the synthesized catalyst here was compared with the reports on the FePc derivatives catalyzed reactions regarding oxidant, temperature, TON, and selectivity (Table 2). It can be found out that in spite of higher TON of previously studied catalyst, they need higher temperatures as well as more expensive and pollutant oxidant. In the meantime, FePc@ChA promotes the reaction without any by-product, which is attributed to milder reaction conditions.

**Table 2:** Comparison of the catalysts performance for the oxidation of BA.

Entry	Catalyst	Oxidant	Temperature (°C)	Selectivity (%)	TON
1	Polyflouro/FePc^26^	TBHP	50	81	582
2	Tetra-substituted FePc^27^	TBHP	70	72	280
3	Flouro-substituted FePc^28^	TBHP	90	80	540
4	FePc@ChA	H_2_O_2_	25	100	111

## Conclusion

FePc was perched on ChA, as a high aspect ratio material, via ionic interactions. The ionic interactions were created as a result of formation ammonium cations on ChA and sulfonate anions on FePc. The simple modification reaction did not impose overhead chemical costs and the environmental pollution concerns. The heterogeneous catalyst evinced an excellent activity in the oxidation of alcohols, and alkyl arenes to the corresponding aldehydes and ketones using H_2_O_2_ as an oxidant during 24 h. The reaction for aromatic alcohols were proceeded at room temperature with the TONs of 106–117, while the aliphatic alcohols were oxidized in harsher conditions at 80 °C with the TONs laying in the range of 106–109. The oxidation of alkyl arenes were needed even higher temperatures, in which TONs of 106–117 were obtained at 100 °C. A comparison of the obtained results with the previous reports demonstrated the efficiency of the presented protocol for the oxidation reactions. We believe that the research provides a new insight about the phthalocyanines loading on the natural-based polymers to synthesis of the efficient heterogeneous catalysts, enjoying from sustainability, easy construction procedure, low costs, and high activity.
